# Control and sensation of breathing during cycling exercise in hypoxia under naloxone: a randomised controlled crossover trial

**DOI:** 10.1186/2046-7648-2-1

**Published:** 2013-01-02

**Authors:** Laurent Koglin, Bengt Kayser

**Affiliations:** 1 Sports Medicine Unit, La Tour Hospital, Meyrin, 1217, Switzerland; 2 Institute of Movement Sciences and Sports Medicine, Faculty of medicine, University of Geneva, 10, rue du Conseil Général, Genève 4, 1205, Switzerland

**Keywords:** Altitude, Exercise, Human, Opioid, Hypoxia

## Abstract

**Background:**

Opioid receptors are possibly involved in the perception of exertion and the ventilatory response to exercise. We compared incremental cycling exercise in conditions of normoxia and hypoxia (11% O_2_) after injection of the opioid receptor antagonist naloxone (30 mg i.v.) or placebo. Naloxone was expected to increase sensation of breathing and cycling and to curtail exercise performance more in hypoxia.

**Methods:**

Ten healthy subjects (29 ± 2 years, 183 ± 6 cm, 75 ± 7 kg, mean ± SD) cycled in normoxia and hypoxia until voluntary exhaustion, receiving naloxone or placebo in a balanced double-blind crossover design.

**Results:**

Hypoxia decreased peak power output by 37%–39% with placebo and naloxone (*P* < 0.001, no effect of naloxone). Switching to normoxia at exhaustion in hypoxia allowed continuing up to 97%–100% of power developed in normoxia with placebo and naloxone (*P* < 0.001, no effect of naloxone). Perceived exertion increased in hypoxia, dropped upon switching to normoxia and increased again towards exhaustion, no effect of naloxone. SpO_2_ (earlobe oximetry) was lower in hypoxia, dropping to 64%–68% with naloxone and placebo. The ventilatory response to exercise in normoxia and hypoxia was not changed by naloxone.

**Conclusions:**

It follows that in healthy subjects the ventilatory response and the perception of exertion in hypoxia as compared to normoxia do not involve the endogenous opioid system, and the latter does not play a role in limiting maximum exercise capacity in hypoxia.

## Background

In both healthy subjects and patients, dyspnoea and leg fatigue are the main symptoms limiting exercise capacity [1-4]. Dyspnoea is accompanied by activation of cortico-limbic structures implicated in interoceptive awareness and nociceptive sensations, such as pain, and involves the opioid system [5]. In patients with dyspnoea, exogenous opioids can alleviate breathing-related discomfort and improve exercise performance [5-8], while injection of naloxone hydrochloride, a non-specific opioid antagonist that crosses the blood–brain barrier, can decrease performance [5]. Opioids can relieve dyspnoea by altering central processing of efferent and afferent sensory information [5]. Sgherza et al. [9] found that in normoxia, in healthy trained subjects, naloxone compared to placebo decreased incremental exercise performance and suggested that sensation of exertion is under influence of endogenous opioids and may be a limiting factor for maximum aerobic exercise capacity.

Acute hypoxia is a potent stressor, especially when combined with an exercise challenge, changing the perceived level of exertion [10]. Acute exposure to hypoxia increases ventilation and cardiac output in order to minimise the reduction in arterial oxygen content and systemic mass oxygen transport. Despite these acute adaptations, incremental exercise testing in such conditions (e.g. an FiO_2_ equivalent to an altitude of 5,000 m or higher, hereafter referred to as severe hypoxia) invariably results in compromised aerobic exercise capacity. The mechanisms behind this limitation of exercise capacity in hypoxia are still poorly understood [11-13]. A puzzling observation is that despite maximum effort in severe hypoxia, cardiac output remains submaximal, suggesting early motor drive withdrawal [11,12]. However, while the locomotor muscles are not driven as hard during large muscle volume effort in such conditions, the contrary is the case for the respiratory muscles. Ventilation is higher for any given level of oxygen consumption during exercise in hypoxia, as compared to normoxia, and is accompanied by a concomitant increase in the sensation of breathing effort [10]. Since blocking the effect of endogenous opioids on the sensation of exertion can increase symptom intensity and curtail performance in normoxia [9], it is possible that such an effect would be exacerbated in hypoxia. We therefore hypothesised that blocking opioid receptors may decrease aerobic exercise performance more in hypoxia than in normoxia. To test this hypothesis, we compared incremental cycling exercise in conditions of normoxia and hypoxia (11% O_2_) after injection of the opioid antagonist naloxone in comparison to a placebo. Our expectations were that under naloxone the sensation of breathing effort and that of cycling would increase and curtail exercise performance more in hypoxia as compared to normoxia.

## Methods

### Subjects

Thirteen healthy trained men volunteered to participate in the study. Inclusion criteria were an age between 18 and 30 years and >4 h/week of endurance training. Exclusion criteria were presence of any relevant chronic or acute disease, having done a maximal capacity training or a race during the last 48 h, symptoms/signs of viral illness in the week preceding the experiments or exposure to altitude in the 2 months before the study. The study was approved by the research ethics commission of the Geneva University Hospitals and SwissMedic and complied with the principles of the Declaration of Helsinki. All subjects were screened by a physician, were fully informed of the nature and risks of the experiments, knew of their right to withdraw at any time and signed an informed consent form.

### Exercise protocol

After inclusion, the subjects first performed two habituation experiments, identical to the real experiments except for the injection, to get used to the equipment and to experience cycling in acute hypoxia. They were instructed not to do any heavy training on the days preceding the experiments and to refrain from caffeine in the 4 h preceding a test. To control for circadian rhythm, for a given subject, all experiments were performed at similar times of the day. After the habituation tests, the subjects came to the lab on four different occasions at least 24 h apart to perform incremental cycling exercise until voluntary exhaustion, twice in normoxia and twice in acute normobaric hypoxia (FiO_2_ = 10.65% O_2_, in Geneva the equivalent of an altitude of approximately 5,000 m). The subjects cycled at 80 rpm on a mechanically braked ergometer (Monark 282E, Varberg, Sweden). After 3 min of resting baseline measurements, the subjects would warm up at 40 watt for 3 min. In order to obtain similar durations of exercise times between subjects and between conditions of normoxia and hypoxia, the duration of steps was adapted between conditions and stages. After warm-up, the subjects incremented by 3-min steps of 40 up to 200 watt in normoxia and then by 20 watt up to voluntary exhaustion, while in hypoxia, they incremented by 40 up to 120 watt and then by 20 watt up to voluntary exhaustion. When reaching exhaustion in hypoxia, the subjects were switched to room air (‘normoxia switch’) and strongly encouraged to continue cycling while the load was increased by 20 watt every 90 s until reaching secondary voluntary exhaustion.

### Intervention

In each condition, they did this once after immediate pre-exercise intravenous injection of naloxone (30 mg naloxone HCl in 30 ml saline) and once after placebo (30 ml saline). The conditions normoxia and hypoxia were in a randomised order. Naloxone and placebo were administered in a balanced double-blind crossover design. The research support section of the Geneva University Hospitals pharmacy prepared the vials, the numbering scheme and the randomization envelopes and released the randomization key after data analysis was completed. The subjects started exercising within 5 min after injection and reached exhaustion within 30 min, approximately one half of the serum half-life of naloxone reported in humans [14].

### Normoxia and hypoxia

Normobaric hypoxia was obtained by mixing N_2_ into ambient air under control of FiO_2_ (Altitrainer, SMTec, Nyon, Switzerland). The gas-mixing system was attached via a piece of large-bore low-resistance tubing to the inspiratory valve of a low-resistance three-way valve (Hans Rudolph 2700, Shawnee, KS, USA) mounted in series with a turbine flow measurement set-up (Vmax 29c, Sensormedics, Loma Linda, CA, USA) attached to a tightly fitted face mask (Hans Rudolph). The subjects always breathed through the same set-up, also in normoxia. The gas-mixing device was set to room air for the normoxia experiments and to a simulated altitude of 5,000 m for the hypoxia experiments.

### Material and measurements

Gas exchange and breathing parameters were measured breath-by-breath with a metabolic cart (Vmax 29c, Sensormedics). Prior to each experiment, the system was calibrated with a 3-L syringe and gas mixtures of known composition. Heart rate was measured by telemetry with a thoracic belt (Polar, Tampere, Finland). Arterial blood haemoglobin saturation (SpO_2_) was measured on an earlobe with a pulse oximeter (Ohmeda, Helsinki, Finland) connected to the metabolic cart. Arterialized blood from a hyperaemic earlobe (Trafuril Cream, Ciba-Geigy, Basel, Switzerland) was used to measure lactate (Accutrend, Roche, West Sussex, UK).

To quantify locomotor muscle activation, a surface electromyogram (EMG) was obtained from the right vastus lateralis muscle. After cleaning with ether and light abrading of the skin, two electrodes (Kendall H59P, Mansfield, OH, USA) were applied directly next to each other on the distal part of the muscle. In addition to marking the skin with indelible ink, we used transparent foil to mark the sites of the electrodes together with skin marks such as moles and scars to reposition the electrodes on the same sites between sessions. Inter-electrode resistance was measured and considered acceptable if <3 kΩ. A reference electrode was placed over a bony area near the knee. The signal was amplified, filtered with a Butterworth band pass between 10 and 200 Hz (BMA-830, CWE, Ardmore, OK, USA), digitised at 1,000 Hz with an AD-board (NI-Daqcard, National Instruments, Austin, TX, USA) and stored on a computer. The data were analysed *post hoc* with custom routines in Matlab (Matlab, Natick, MA, USA) to obtain, for each single contraction, the integrated rectified EMG (iEMG) and, after a fast-Fourier transformation, the median (i.e. centroid, CPF) and mean power (MPF) frequencies, as described before [15]. iEMG was normalised with the signal obtained at 80 watt.

### Perception of exertion

The subjects were asked to rate the rate of perceived exertion (RPE) on a 0–10-point CR-10 Borg ratio scale [2]. At the end of each exercise level, the subjects rated their perception of exertion separately for their legs (How hard is it to cycle?), breathing (How hard is it to breathe?) and overall (How hard is the overall effort?). The anchors were 0 for no exertion at all and 10 for the maximum imaginable. When prompted, the subject would point to the scale and nod when the experimenter called the correct corresponding number out loud.

### Analysis and statistics

For each subject and each condition, the data were averaged over the last 30 s of each workload with the exception of the maximum when a mean over 15 s was used. The data were analysed with SPSS version 18 (IBM, Chicago, IL, USA). Repeated measures ANOVA was used to test for within-group effects across time. Following significant main effects, planned pairwise comparisons were made using Holm's sequential Bonferroni procedure. Results are expressed as mean ± SD. Statistical significance was set at *P <* 0.05.

## Results

Of the 13 recruited subjects, 1 dropped out after the first habituation test (no reason given), 2 subjects were excluded for a vagal reaction to hypoxia at rest, and 10 completed the study (age 29 ± 2 years (mean ± SD), height 183 ± 6 cm, weight 75 ± 7 kg, maximum aerobic capacity (V'O_2_max) 50 ± 8 ml/kg/min). The injection of naloxone was well tolerated. Table 1 shows the results observed at exhaustion in the different conditions.

**Table 1:** Peak values at exhaustion in normoxia, hypoxia and after the normoxia switch at exhaustion in hypoxia

	**Normoxia**		**Hypoxia**		**Normoxia switch after hypoxia**	
	**Placebo**	**Naloxone**	**Placebo**	**Naloxone**	**Placebo**	**Naloxone**
Power (watt)	296 ± 48	292 ± 52	182 ± 36^*^	184 ± 34^*^	282 ± 51	291 ± 49
Time (min)	22.5 ± 2.6	22.3 ± 2.4	22.0 ± 3.8	22.4 ± 3.7	27.1 ± 4.2	27.6 ± 3.9
HR (/min)	184 ± 5	181 ± 7	169 ± 7^*^	168 ± 7^*^	173 ± 5	170 ± 6
Lactate (mM)	11.3 ± 3.7	10.5 ± 2.4	10.4 ± 2.9	10.3 ± 3.7	11.3 ± 3.0	10.9 ± 5.1
RPE global (a.u.)	9.7 ± 0.5	9.6 ± 0.5	9.4 ± 0.7	9.7 ± 0.5	9.3 ± 0.9	9.4 ± 0.5
RPE resp (a.u.)	9.5 ± 0.7	9.4 ± 0.9	9.5 ± 0.6	9.5 ± 0.7	9.0 ± 1.1	8.9 ± 0.4
RPE legs (a.u.)	9.9 ± 0.3	9.7 ± 0.7	9.6 ± 0.5	9.8 ± 0.4	10.0 ± 0.0	10.0 ± 0.0
SaO_2_ (%)	91.7 ± 4.1	93.3 ± 6.4	67.5 ± 8.9^*^	63.6 ± 8.8^*^	90.55±	95.70±
P_ET_CO_2_ (kPa)	4.44 ± 0.70	4.56 ± 0.48	3.50 ± 0.18^*^	3.49 ± 0.26^*^	4.48 ± 0.32	4.42 ± 0.40
V'O_2_ (L/min)	3.77 ± 0.8	3.85 ± 0.76	2.39 ± 0.40^*^	2.39 ± 0.40^*^	3.53 ± 0.71	3.64 ± 0.63
V'CO_2_(L/min)	4.92 ± 1.1	4.86 ± 0.80	3.19 ± 0.53^*^	3.30 ± 0.57^*^	3.86 ± 0.78	4.05 ± 0.85
V'_E_ (L/min)	150 ± 33	146 ± 21	128 ± 20^*^	133 ± 24^*^	123 ± 21	132 ± 28
V'_A_ (L/min)	159 ± 43	154 ± 27	133 ± 26^*^	137 ± 37^*^	126 ± 27	138 ± 37
Vt (L)	2.98 ± 0.39	3.16 ± 0.41	2.89 ± 0.51	2.97 ± 0.72	2.91 ± 0.45	3.09±
RR (/min)	50 ± 8	47 ± 7	45 ± 7	44 ± 7	42 ± 6	43 ± 5
MEFR (L/sec)	5.1 ± 1.2	5.0 ± 0.8	4.2 ± 0.7	4.3 ± 0.9	4.1 ± 0.8	4.4 ± 1.0

Naloxone had no effect on any of these parameters in either condition. Power, mechanical power output on cycle ergometer; time, time to exhaustion; HR, heart rate; lactate, arterialized lactate concentration; RPE, rate of perceived exertion (overall, respiratory, legs); SaO_2_, earlobe oximetry; P_ET_CO_2_, end-tidal CO_2_; V'O_2_, oxygen consumption; V'CO_2_, expired CO_2_; V'_E_, minute ventilation; V'_A_, alveolar ventilation; Vt, tidal volume; RR, respiratory frequency; MEFR, peak expiratory flow. ^*^Significantly different from normoxia.

Hypoxia decreased power output by 39% in the placebo condition (*P* < 0.001) and by 37% in the naloxone condition (*P* < 0.001, no significant difference between conditions). Switching to normoxia at exhaustion in hypoxia allowed continuing up to 97% of power developed in normoxic control under placebo (*P* < 0.001) and to almost 100% under naloxone (*P* < 0.001, no significant difference between conditions).

V'O_2_ peak was 3.77 ± 0.80 L/min (placebo) and 3.85 ± 0.76 L/min (naloxone) in normoxia, reached 2.39 ± 0.4 L/min in hypoxia (both placebo and naloxone) and increased again to 94% (placebo) and 95% (naloxone) of normoxia values after the normoxia switch at exhaustion from hypoxia (no significant difference between conditions). Time to exhaustion was similar in normoxia and hypoxia (22 ± 3 min) and reached a total of 27 ± 4 min for hypoxia when adding the additional exercise time after the normoxia switch (no effects of naloxone).

**Figure 1:**
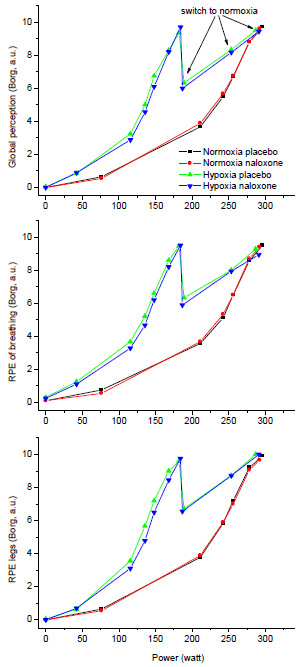
**The *top panel *shows the overall RPE vs. power output.** At exhaustion from exercise in hypoxia, the subjects were switched to room air and strongly encouraged to continue pedalling until reaching secondary exhaustion. The *arrows* indicate the measurements after the switch; for reasons of legibility, the *arrows* are not shown on the other graphs. The *middle panel* shows the RPE breathing vs. power output. The *bottom panel* shows the RPE legs vs. power output. Error bars were omitted for clarity. The coloured symbols and lines represent the same conditions for all figures.

Figure 1 shows the overall levels of perceived exertion and those pertaining to breathing and leg effort separately. In normoxia, perceived exertion increased in a curvilinear way, and there was no effect of naloxone. The rate of increase was greater in hypoxia, dropped upon switching to acute normoxia at exhaustion, and increased again towards exhaustion (no effect of naloxone).

**Figure 2:**
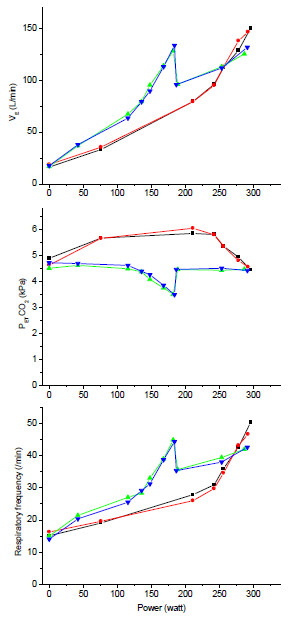
**The *top panel *shows the total ventilation (V'E) vs. power output.** The *middle panel* shows the end-tidal CO_2_ tension (P_ET_CO_2_) vs. power output. The *bottom panel* shows the respiratory frequency vs. power output.

The top panel of Figure 2 shows the increase in minute ventilation (V'_E_) with exercise intensity and its more pronounced increase in hypoxia. After the normoxia switch, ventilation dropped but not completely to the normoxic level. There was no effect of naloxone. The second panel of Figure 2 shows the evolution of P_ET_CO_2_. In conditions of normoxia, the typical pattern of a slight increase followed by a drop beyond the ventilatory threshold was observed. Conversely, hypoxia immediately induced hyperventilation and reduced P_ET_CO_2_ values, which were not restored upon the normoxia switch. There was no effect of naloxone. The third panel of Figure 2 shows the evolution of respiratory frequency, which followed a similar pattern as that of ventilation and tidal volume (not shown), without any effect of naloxone.

**Figure 3:**
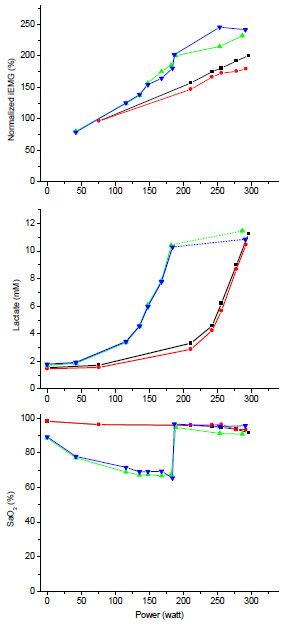
**The *top panel *shows normalised integrated vastus lateralis surface electromyogram (iEMG) vs. power output.** The *middle panel* presents the arterialized blood lactate concentration [La] vs. power output. The *bottom panel* presents the arterial oxygen saturation (earlobe oximetry) vs. power output.

iEMG was higher during hypoxia (Figure 3). No differences were observed in CPF or MPF (not shown). At exhaustion in hypoxia, after the hypoxia switch, iEMG increased and reached higher values at exhaustion compared to normoxia. There were no effects of naloxone.

Blood lactate levels (Figure 3) increased in a typical curvilinear manner both in normoxia and hypoxia, with an early onset of the exponential increase in hypoxia. There was no effect of naloxone.

SpO_2_ showed a slight drop at higher intensities in conditions of normoxia, whereas it dropped right from the start of exercise in hypoxia. Upon the normoxia switch, it normalised rapidly. There was no effect of naloxone. SpO_2_ reached lower values in hypoxia, dropping to 68% with placebo and 64% with naloxone (no significant difference), and increased after the normoxia switch. Heart rate response to exercise showed the typical linear increase with a steeper slope in hypoxia, a drop at the normoxia switch and similar maximum heart rates at exhaustion (no effects of naloxone; data not shown).

**Figure 4:**
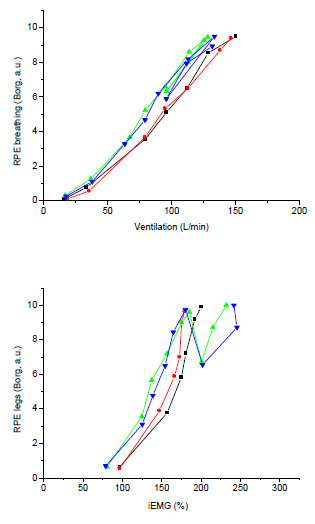
**The *top panel *shows RPE breathing vs. total ventilation.** The *bottom panel* shows RPE legs vs. iEMG.

Figure 4 shows the relationship between ventilation and RPE breathing, and iEMG and RPE legs. Both relationships were slightly displaced to the left in hypoxia compared to normoxia, but there was no effect of naloxone.

## Discussion

Contrary to our expectations naloxone had no effect on any of the investigated variables, neither in normoxia nor in hypoxia. It follows that, at least in trained healthy young male subjects, during incremental exercise tests, in normoxia and normobaric hypoxia with an FiO_2_ of 10.65 (equivalent to approximately 5,000 m), endogenous opioid receptors are not involved in the ventilatory and heart rate responses to exercise nor in the sensation of overall levels of perceived exertion or those pertaining to breathing or cycling effort specifically.

### Naloxone dosage

Could it be that there was insufficient blockade of opioid receptors? This seems unlikely. The ‘normal’ dose for clinical use of naloxone is 1–4 mg, largely sufficient for full reversal of the effects of exogenously administered opioids and to trigger withdrawal symptoms [16]. Naloxone hydrochloride is partly actively transported through the blood–brain barrier and reaches higher central nervous system (CNS) concentrations than in the plasma [17]. Positron emission tomography studies showed that with 1 mg naloxone, 50% of opioid receptors in the CNS were blocked [18]. Santiago and Edelman [19] recommended a minimal dose of 0.1 mg/kg for peripheral and central receptor blockade. We used 30 mg, i.e. 0.40 ± 0.04 mg/kg, a dose that is four times in excess, to compare our results to those of a previous study [9]. Naloxone has a half-life of about 1 h [14]. Our subjects started exercising within 5 min after injection and reached exhaustion in less than 30 min, largely inside the therapeutic time window. Finally, even though admittedly anecdotal, several subjects indicated missing their habitual post-exercise ‘high’ and feeling somewhat ‘bland’ instead, which after the breaking of the randomization key appeared to correspond to the naloxone experiments, suggestive of naloxone blocking the effect of endogenous opioid release post-exercise, possibly related to the ‘feeling good’ after a workout [20,21].

### Opioids and dyspnoea

What can explain the difference between our findings and the repeated finding of a role for opioids in patients with dyspnoea? For example, naloxone increased dyspnoea sensation in asthmatics when challenged with metacholine, and similar findings were reported for chronic obstructive pulmonary disease (COPD) patients during exercise [22,23]. Jensen et al. [7] compared inhaled nebulized fentanyl citrate (an opioid analogue) to placebo in COPD patients and found dyspnoea attenuation and improved exercise performance. Both sensations of intensity and unpleasantness were affected. There are similar observations in patients with terminal cancer-related dyspnoea [24], and opioids have a role as therapeutic means in dealing with dyspnoeic patients in general [5,6,25].

One explanation for an absence of effect in our study may reside in the complexity of what is covered by the term dyspnoea. The American Thoracic Society defined dyspnoea as ‘a subjective experience of breathing discomfort that consists of qualitatively distinct sensations that vary in intensity’ [5,26]. Dyspnoea includes both sensory (intensity) and affective (unpleasantness) components [26]. Distinct mechanisms and afferent pathways are associated with different sensory qualities (notably work/effort, tightness and air hunger/unsatisfied inspiration); distinct sensations most often do not occur in isolation, and dyspnoea sensations also vary in their unpleasantness and in their emotional and behavioural significance [5]. In patients, the affective component is often of great importance [27], whereas it is of less significance in a healthy athletic subject during a non-threatening challenge like an exercise test limited in time. A patient with COPD associates the difficulty to breathe during an exercise test with fear to suffocate, whereas a healthy subject will not [28]. Nevertheless, in healthy subjects, intravenous morphine sulphate reduces the discomfort (‘air hunger’) induced by hypercapnia to a similar extent as observed during clinical studies [25].

Breathing during heavy exercise in severe hypoxia is accompanied by dyspnoea with affective aspects [29]. In the present study, the subjects quantified the rate of perceived exertion in the classic sense originally proposed by Borg [2], i.e. the central generated sensation of effort [30]. Affective components of unpleasant sensations related to the exercise like leg pain or air hunger were not explicitly quantified. There are two different dimensions of exercise-related sensations: (1) one reflecting the sensation of effort, likely centrally generated and unrelated to afferent feedback and (2) one related to afferent feedback from various tissues involved in the effort [30]. Aliverti et al. [10] reported that breathing RPE was uniquely related to total respiratory power output at low and high altitudes (hypobaric hypoxia, 4,559 m). In the present study, in conditions of normobaric hypoxia, the tendency for a leftward shift of the curve relating breathing RPE to V'_E_ (see Figure 1) suggests that breathing RPE may have been influenced by other parameters but that the endogenous opioid system was not involved. However, even if an affective component of the sensation of breathing was influenced by hypoxia and/or naloxone in our study, this did not have an effect on performance.

### Ventilatory response to exercise

Anatomical and pharmacological evidence suggests that endogenous opioids play a role in the control of breathing. Mu (μ), delta (δ) and kappa (κ) opioid receptors are present in brainstem areas involved in respiration, and endogenous opioids like endorphins, enkephalins, dynorphins and endomorphins are found in medullary and pontine respiratory regions [6,31]. The depressant effects of exogenous opioids on ventilation are well known, and also, endogenous opioids are thought to be tonically active and have a depressant effect on ventilation [31]. Exogenous opioids also have a strong depressant effect on the hypoxic ventilatory response in animals and humans [32]. By contrast, on a local CNS level, endogenous opioids may exert an excitatory modulation of hypoxia-induced hyperventilation by acting on μ-receptors in the rostral medullary raphe at least in a rat model [33], illustrating that the overall systemic effects of exogenous opioids are not necessarily indicative of the role of endogenous opioids at specific sites in the CNS. Akiyama et al. [34] injected healthy subjects with naloxone hydrochloride and found an increase of both ventilatory (V'_E_) and mouth pressure (Pm) responses to hypoxic progressive hypercapnia with inspiratory flow-resistive loading. Ward and Nitti [35] injected the opioid agonist sufentanil in trained athletes during exercise and found that the ventilatory response to exercise was reduced. We expected that the injection of 30 mg of naloxone would increase the ventilatory response to exercise. Our results indicate no effect of a general blockade of opioid receptors on the ventilatory response to incremental exercise in normoxia and hypoxia. It follows that it is unlikely that endogenous opioids play any important role in the ventilatory response to an incremental exercise challenge in normoxia and its increase in hypoxia, at least in healthy, young trained male subjects.

### Central effects

Humans frequently report positive feelings during and after endurance efforts (‘runner's high’) that include both central effects (improved affect, sense of well-being, anxiety reduction, post-exercise calm) and peripheral effects (reduced pain sensation) [21]. Imaging studies provided evidence of endogenous opioid release in fronto-limbic brain regions after physical exercise correlated to perceived euphoria [21]. Endocannabinoids are also thought to play a role in runner's high [36]. Paulev et al. [37] compared naloxone (0.8 mg i.v.) to placebo during a Cooper test (running the longest possible distance within 12 min) in trained subjects. Performance was not influenced by naloxone, but perception of muscle pain was enhanced with naloxone. Sgherza et al. [9] compared incremental exercise capacity in 18 subjects under naloxone and placebo and reported a significant reduction with naloxone. They concluded that after naloxone administration, in laboratory conditions, endurance trained subjects stop exercise at lower levels than those under placebo, suggesting that peak exercise capacity was limited by the individual's perception of exertion, exacerbated by a lack of effect from endogenous opioids, rather than by physiological fatigue. Even though several subjects in our study reported feeling ‘bland’ after exercise under naloxone, we did not see the reduction in exercise capacity reported by Sgherza et al. [9] in normoxia. Their results were based on 18 subjects, and earlier studies with fewer subjects and lower dosage of naloxone had failed to find significant effects. We therefore cannot exclude that our results, obtained in ten subjects, are limited by type-II error, but given the absence of any change in the variables monitored, it seems quite unlikely that in hypoxia an effect of opioid blockade plays any important role.

### Leg afferents

Apart from their involvement in noxious signalling, spinal cord level opioid receptors may play a role in the exercise pressor response. Amann et al. [38-40] used lumbar intrathecal fentanyl to block central projection of μ-opioid-receptor-sensitive group III/IV muscle afferents from the lower limbs during different exercises: single-leg mild to moderate intensity knee extensor exercise, 5-km cycling time trial exercise, incremental cycling exercise and constant load exercise to exhaustion. Afferent blockade had no effect on central and peripheral haemodynamics or ventilation at rest, but during exercise cardiac output, mean arterial pressure and femoral blood flow were attenuated in the knee extensor exercise, pacing during the time trial was severely perturbed, and heart rate, blood pressure and ventilation were impeded during cycling at 80% of aerobic maximum. These findings suggest an important role of group III/IV muscle afferents in the regulation of the cardiorespiratory response to rhythmic exercise. In rats, Tsuchimochi et al. [41] found that stimulation of peripheral μ-opioid receptors attenuated the exercise pressor reflex and that this effect could be blocked with naloxone. These findings in humans and animals would suggest that endogenous opioids play a role in the regulation of muscle afferent traffic, but from our results, it appears that in healthy, young, physically active subjects doing a 20- to 30-min incremental exercise challenge in normoxia or severe hypoxia, endogenous opioids are not attenuating the normal cardiovascular response to exercise through the stimulation of type III/IV afferent μ-receptors at the spinal level, leaving the question on their role and significance open.

### Acute normoxia switch

The limitation of exercise performance in severe hypoxia is still not well understood [11-13]. Lack of oxygen is the reason, but it remains unclear what mechanism in hypoxia leads to an earlier disengagement from an exercise challenge as compared to normoxia. Verges et al. [13] argue that because biochemical, electromyographic and mechanical signs of muscle fatigue at exhaustion are reduced in severe hypoxia compared with normoxia, muscle metabolic fatigue is not the main factor responsible for impaired whole body exercise performance, as proposed before [12,15]. Our subjects were able to continue cycling after reaching their maximum in hypoxia when switched to normoxia. iEMG, an indicator of locomotor muscle drive, increased upon the normoxia switch, a finding arguing in favour of supraspinal limitation of exercise performance in hypoxia by a rapidly reversible withdrawal of motor drive. Amann et al. [42] found that the degree of hypoxia influences the relative role of muscle fatigue in the cessation of dynamic exercise with large muscle groups. Those authors proposed a threshold of SaO_2_ for a switch from a predominant effect of peripheral fatigue to a predominant effect of CNS hypoxia on central motor output and exercise performance. They found similar levels of muscle fatigue in task failure from constant load exercise to exhaustion when SaO_2_ averaged 94%, 82% or 76% (FiO_2_ 0.21–0.12), but not at a SaO_2_ of 67% (FiO_2_ 0.10). They suggest the dominance of CNS hypoxia over peripheral muscle fatigue in influencing central motor output below SaO_2_ levels of 70%–75%. Our results of early exhaustion in hypoxia with SaO_2_ below 70% and rapid restoration of exercise capacity upon the normoxia switch are in accordance with those contentions.

### Limits of performance in severe hypoxia

The origins of the signals leading to the cessation of the central motor drive at exhaustion during heavy large muscle group exercise in severe hypoxia thus remain unclear. Possible candidates controlling the signal to stop exercise include arterial oxygen (O_2_) desaturation with exercise causing marked central nervous system hypoxia, other factors acting on the respiratory and/or other higher nervous centres, with or without contribution of fatigued respiratory muscles, or the effects of pulmonary hypertension and right ventricular overload [4,12,43,44]. Millet et al. [45] showed in a biceps brachii repeated isometric contraction model that in pronounced hypoxia (9% O_2_, SaO_2_ 75%), central drive is diminished independently of afferent feedback and peripheral fatigue and concluded that submaximal performance in severe hypoxia is related directly to brain oxygenation, results corroborating those of Goodall et al. [46] who also showed that peripheral mechanisms of fatigue contribute relatively more to the reduction in force-generating capacity of the knee extensors following submaximal intermittent isometric contractions in normoxia and mild to moderate hypoxia, whereas supraspinal fatigue plays a greater role in more pronounced degrees of hypoxia.

### Study limitations

Our results pertain to a limited sample size of healthy, young, trained male subjects and cannot be generalised. Further limitations to our study design are acknowledged. Prolonged hypoxia leads to ventilatory acclimatisation changing ventilation at rest and during exercise. We performed our experiments in acute normobaric hypoxia, and it is possible that in chronic hypobaric hypoxia, findings would differ. It therefore remains to be described what the effects of opioids or opioid receptor antagonists on breathing sensation and exercise performance in conditions of prolonged hypobaric hypoxia would be. Finally, we used a short incremental exercise protocol to exhaustion. It remains an open question if more prolonged submaximal time trial like exercise would be influenced by opioid receptor blockade.

## Conclusions

We compared incremental cycling exercise capacity in conditions of normoxia and hypoxia after injection of the opioid antagonist naloxone, in comparison with placebo. Since naloxone had no effect on the ventilatory response to exercise nor on the sensation of exertion, neither in normoxia nor in hypoxia, it follows that endogenous xopioid receptors do not play a role in the perceived exertion and the regulation of maximal aerobic exercise performance in conditions of severe hypoxia.

## Competing interests

The authors declare that they have no competing interests.

## Authors’ contributions

BK and LK designed the experiment and collected data. LK did the data analysis under the supervision of BK. Both authors wrote the manuscript. BK is the guarantor. Both authors read and approved the final manuscript.
